# Supplementary material for: Pd/NiMoO4/NF electrocatalysts for the efficient and ultra-stable synthesis and electrolyte-assisted extraction of glycolate
Source: Nat Commun. 2024 Apr 4;15:2899. doi: 10.1038/s41467-024-47179-7 (PMC10995147; doi:10.1038/s41467-024-47179-7)
Supplement: Supplementary file 1 — Supplementary Information [file 41467_2024_47179_MOESM1_ESM.docx]

*Supporting Information* *for*

**Pd/NiMoO_4_/NF electrocatalysts for the efficient and ultra-stable synthesis and electrolyte-assisted extraction of glycolate**

Kai Shi^1^, Di Si^1^, Xue Teng^1^, Lisong Chen^1,2*^, Jianlin Shi^3^

^1^Shanghai Key Laboratory of Green Chemistry and Chemical Processes, State Key Laboratory of Petroleum Molecular and Process engineering, School of Chemistry and Molecular Engineering, East China Normal University, Shanghai 200062, China

^2^Institute of Eco-Chongming, Shanghai 202162, China

^3^Shanghai Institute of Ceramics, Chinese Academy of Sciences, Shanghai 200050, P. R. China

*Corresponding author: lschen@chem.ecnu.edu.cn (L. Chen)

**Supplementary Note 1**

To evaluate the economic potential of renewable electricity powered generation of sodium glycolate from ethylene glycol, a techno-economic analysis was carried out. **Supplementary Figure S1** describes the framework, which is used to calculate the total plant gate levelized cost of production with units of US$ per ton of sodium glycolate. In this framework, the costs are divided into two parts, namely the capital costs and the operating costs. Capital costs mainly refers to the electrolyser and solid/liquid separation equipment cost. Operating costs mainly includes four components: electricity, product separation, plant operation, and material (the price of ethylene glycol and sodium hydroxide).


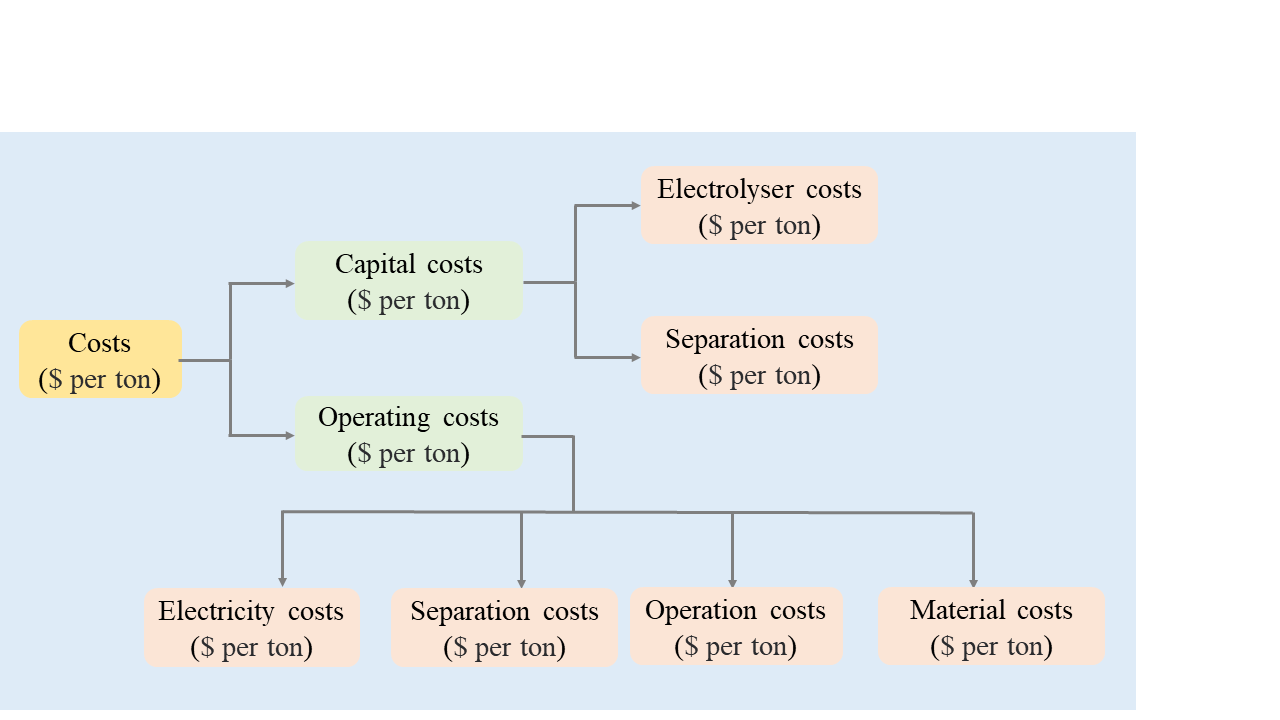


**Supplementary Fig. 1 TEA analysis.** Framework of the techno-economic analysis.

Below, we assume the operating current density of 100 mA cm^-2^ for techno-economic analysis.

1. The costs of electrolyser is assumed to be $10,000 per m^2^ of and the lifetime of plant is assumed to be 10 years.

2. Separations equipment capital costs will be set as 10 % of the electrolyser capital costs.

3. The price of electricity is considered to be 0.1 $/kWh.

4. Separation costs is set as 30% of electricity costs.

5. Operation costs is set as 10% of the capital costs.

6. The plant is assumed to be operational 19.2 hours a day.

7. The faradaic efficiency to sodium glycolate is 80%, the total cell operating voltage is 1.2 V and the operating current density is 100 mA cm^-2^.

7. The plant will convert 200 tons of ethylene glycol and 129 tons of sodium hydroxide to 316 tons of sodium glycolate per day. We assume that 90% of the starting ethylene glycol and sodium hydroxide eventually converts to sodium glycolate; this therefore consumes 400 tons of ethylene glycol and 258 tons of sodium hydroxide per day.

8. As for material costs, the price of ethylene glycol, sodium hydroxide and sodium glycolate is assumed to be $676 per ton, $735 per ton, and $2941 per ton, respectively.

9. The price of hydrogen is set as $1,900 per ton. The faradaic efficiency for hydrogen production is set as 100 %.

Next, we are going to calculate each of the 5 components for a day of operation.

**Material costs**

$$Material costs=\frac{\begin{aligned} Cost of ethylene glycol*Mass of ethylene glycol needed+Cost of sodium hydroxide \\ *Mass of sodium hydroxide needed \end{aligned}}{mass of sodium glycolate produced}=\frac{\$676*400+735*258}{316}=\$1456 per ton sodium glycolate$$

**Electricity costs**

Firstly, we calculate the total charge needed to oxidize 200 tons of ethylene

glycol per day.

$$Q=\frac{Mass of ethylene glycol converted*F*N}{Molar mass of ethylene glycol*faradaic efficirncy}$$

$$=\frac{200*{10}^{6}*96485*4}{62.068*0.8}$$

$$=1.55*{10}^{12}C$$

Where Q is the total charge, F is the Faraday’s constant and N takes the value 4 since ethylene glycol oxidation to sodium glycolate is a four-electron transfer process.

We now calculate the current needed to maintain this process at a capacity factor of 0.8.

$$I=\frac{Q}{Time in a day*Capacity factor}$$

$$=\frac{1.55*{10}^{12}}{24*60*60*0.8}$$

$$=2.24*{10}^{7}A$$

Where *I* is the current.

The power needed to maintain this process can be calculated, assuming an operating cell potential of 1.2 V.

$$P=2.24*{10}^{7}*1.2=2.688*{10}^{4}kW$$

The energy consume per day can be depicted as follows:

$$Energy \mathrm{consume} per day=P*Time in a day*Capacity factor$$

$$=2.688*{10}^{4}*24*0.8=5.2*{10}^{5}kWh$$

Finally, the daily electricity cost normalized by the mass of sodium glycolate produced can be calculated:

$$Electricity cost per day=\frac{Energy use per day*Cost per kWh}{Mass of sodium glycolate produced}$$

$$=\frac{5.2*{10}^{5}*0.1}{316}=\$165 per ton of sodium glycolate$$

**Separation costs**

The separation costs is set as 30% of electricity costs. Hence:

$$Separation costs=\$165*0.3=\$49.5$$

**Capital costs**

Firstly, we calculate the electrolyser cost. The area of electrolyser can be calculated as follows (at the current density of 100 mA cm^-2^ (0.1 A cm^-2^):

$$Area of electrolyser=\frac{2.24*{10}^{7}}{0.1}=2.24*{10}^{8}{cm}^{2}=2.24*{10}^{4}m^{2}$$

The costs of electrolyser is assumed to be $10,000 per m^2^, so the electrolyser cost can be calculated as follows:

$$Cost of electrolyser=2.24*{10}^{4}*10000=\$2.24*{10}^{8}$$

Separations equipment capital costs will be set as 10 % of the electrolyser capital costs. So the capital costs can be calculated as follows:

$$Cost of separations equipment=\$2.24*{10}^{8}*0.1=\$2.24*{10}^{7}$$

As a result, the capital costs component can be calculated as follows:

$$Capital costs=\frac{Cost of electrolyser+Cost of separations equipment}{Lifetime of plant*Mass of sodium glycolate produced}$$

$$=\frac{\$2.24*{10}^{8}+\$2.24*{10}^{7}}{10*365*316}=\$214 per ton sodium glycolate$$

**Operation costs**

Operation costs is set as 10% of the capital costs. So the operation costs can be calculated as follows:

$$Operation cost per day=\$214*0.1=\$21.4$$

**The total costs:**

$$Total costs=\$1456+\$165+\$46.5+\$214+\$21.4=1902.9 per ton sodium glycolate$$

**Potential profit:**

The daily profit can be calculated based on the market price of sodium glycolate.

$$Profit per ton sodium glycolate=\$2941-\$1902.9$$

$=\$1038.1 per ton sodium glycolate$

$$Profit per day=\$1038.1*316=\$328039.6$$

The faradaic efficiency for hydrogen production is set as 100 %.

$$Mass of hydrogen produced per day=\frac{Q*Molar mass of hydrogen}{N*F}=\frac{1.55*{10}^{12}*2}{96485*2}=1.61*{10}^{7}g=16tons$$

The profit of hydrogen can be calculated as follows:

$$Profit per day=\$1900*16=\$30400$$

$$Profit from hydrogen per tons sodium glycalate=\frac{\$30400}{316}=\$96.20$$

**The total profits:**

$$Total profits=\$1038.1+\$96.20=\$1134.3 per ton sodium glycolate$$

**Breakeven point:**

$$Breakeven point=\$2941+\$96.20=\$3037.2$$

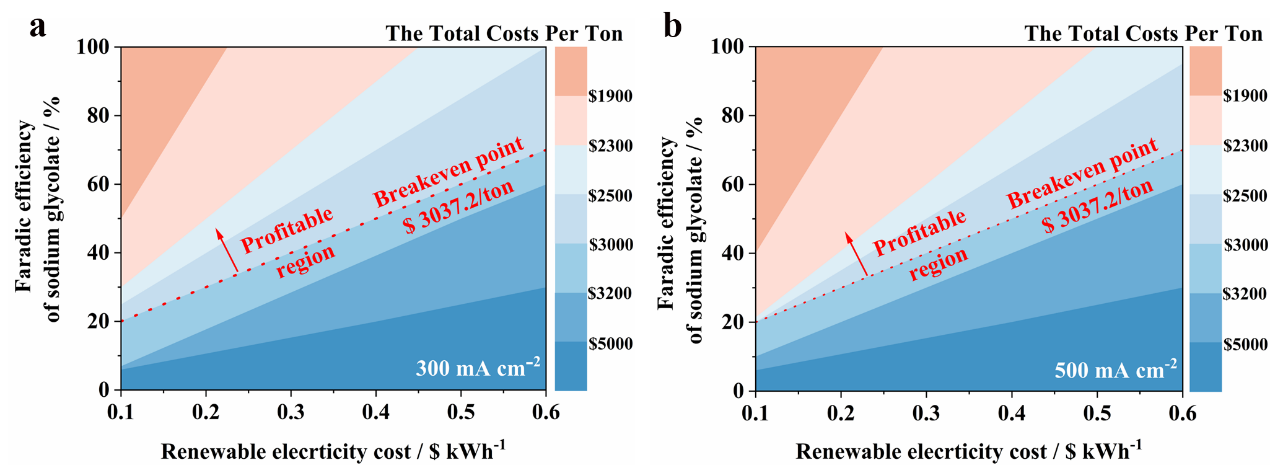


**Supplementary Fig. 2 TEA results at different current densities.** **a** 300 mA cm^-2^, **b** 500 mA cm^-2^. The area above the white dashed line represents the profitable production cost of sodium glycolate.

**Supplementary Note 2**

The SEM images indicate a 3D porous structure of the pure NF with a smooth surface.


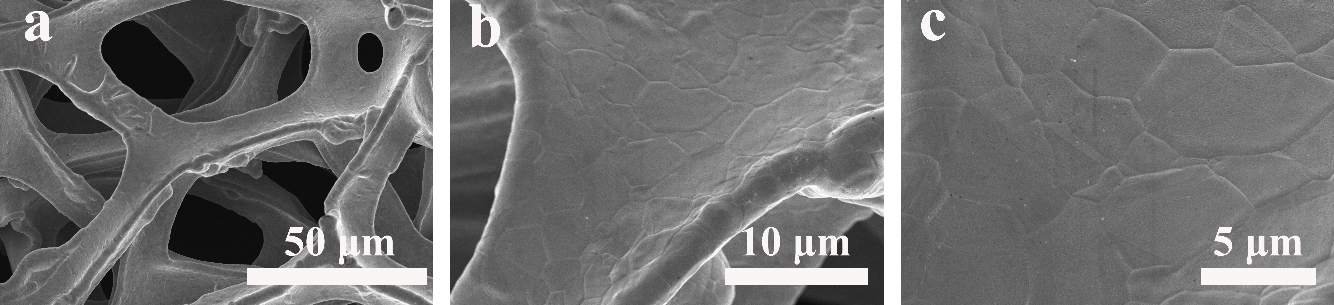


**Supplementary Fig. 3 SEM images of pure NF with different scales.** **a** 50 μm. **b** 10 μm. **c** 5 μm.

**Supplementary Note 3**

The SEM images of the NiMoO_4_/NF indicate that the nanorods of NiMoO_4_ successfully grew on the NF substrate.


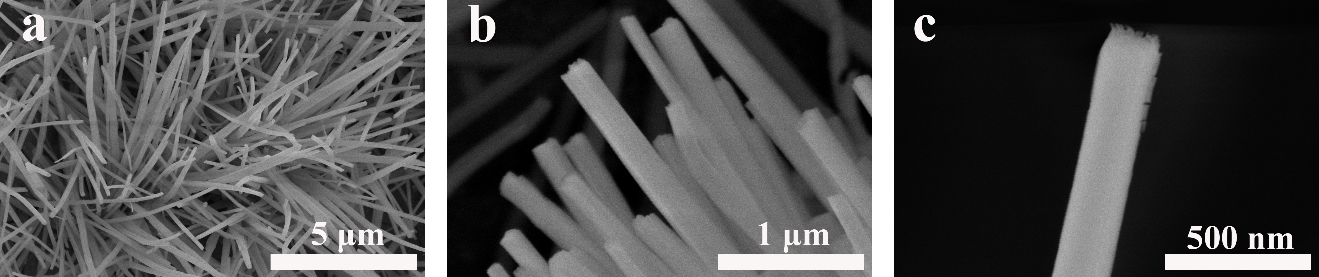


**Supplementary Fig. 4 SEM images of NiMoO_4_/NF with different scales.** **a** 5 μm. **b** 1 μm. **c** 500 nm.

**Supplementary Note 4**

The SEM images of the Pd/NF indicate that the nanosheets of Pd successfully grew on the NF substrate.


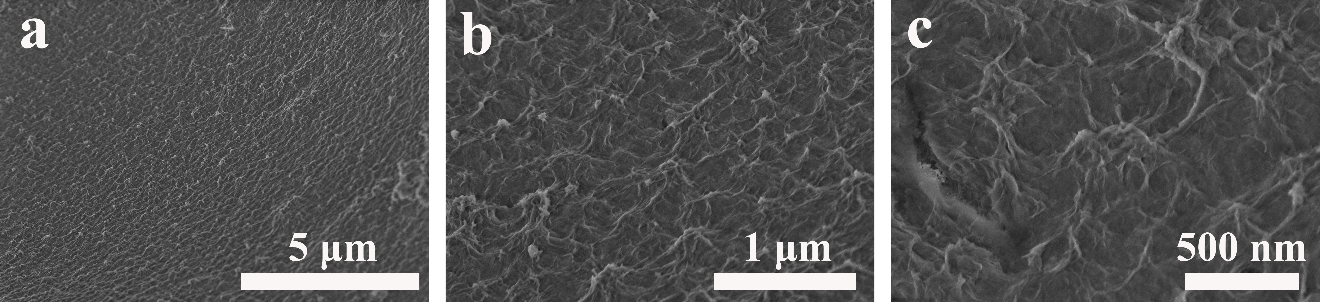


**Supplementary Fig. 5** SEM images of Pd/NF with different scales. **a** 5 μm. **b** 1 μm. **c** 500 nm.

**Supplementary Note 5**

The nanorod structure of NiMoO_4_ has been exhibited in the transmission electron microscopy (TEM) image (**Supplementary Figure 6a**). The distinct lattice fringe of 3.51 Å in the lattice d-spacing is highlighted, corresponding to the ($\bar{\text{1}}\text{12}$) plane of NiMoO_4_, in the high-resolution TEM (HRTEM) images (**Supplementary Figure 6b** insert), which confirmed the crystalline NiMoO_4_. This ($\bar{\text{1}}\text{12}$) plane has been further demonstrated in the reflection located at 25.3° of the X-ray diffraction (XRD) pattern, shown in **Figure 2b**. Besides, the nanosheet structure of Pd has been exhibited in the transmission electron microscopy (TEM) image (**Supplementary Figure 6c**). The distinct lattice fringe of 2.24 Å in the lattice d-spacing is highlighted, corresponding to the (111) plane of Pd, in the high-resolution TEM (HRTEM) images (**Supplementary Figure 6d** insert), which confirmed the crystalline Pd. This (111) plane has been further demonstrated in the reflection located at 25.3° of the X-ray diffraction (XRD) pattern, shown in **Figure 2b**.


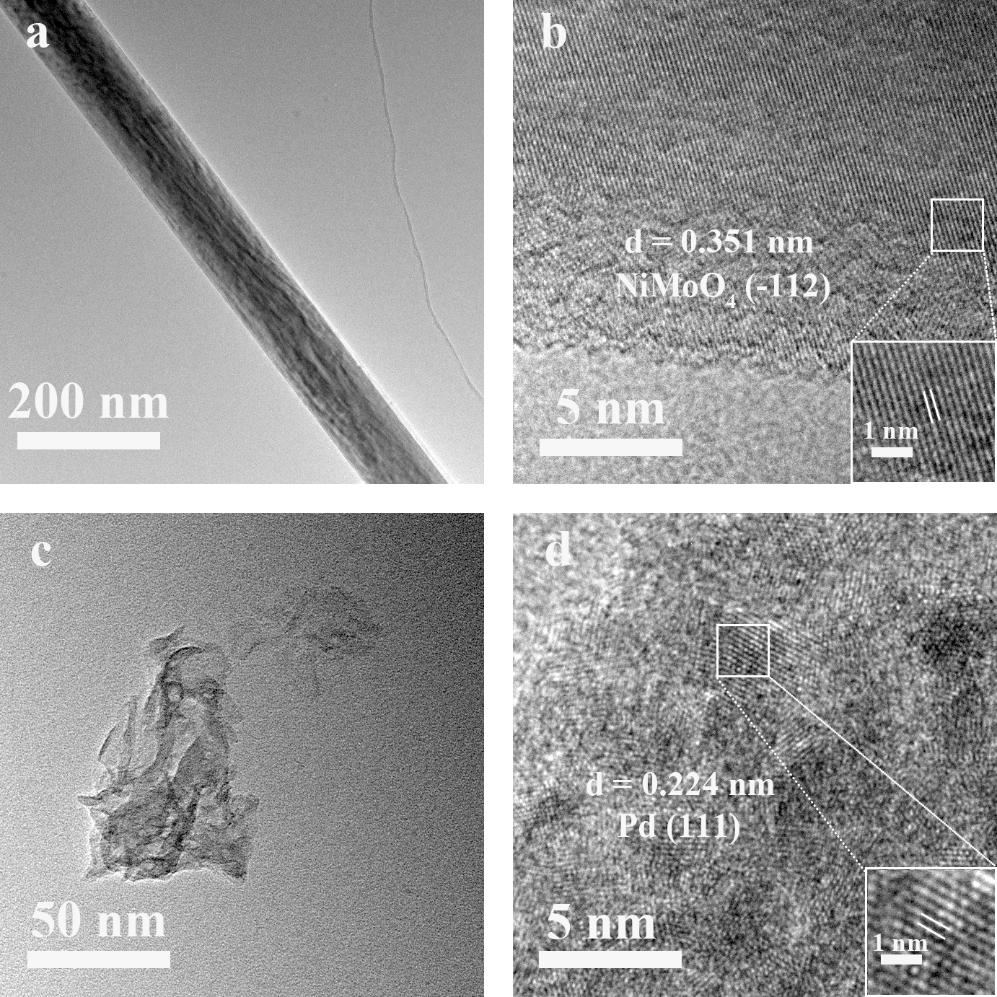


**Supplementary Fig. 6 TEM images.** The **a** TEM and **b** HRTEM images of NiMoO_4_/NF. The **c** TEM and **d** HRTEM images of Pd/NF.





**Supplementary Fig. 7** **The comparision of nobel-metal content.** A nobel-metal content comparison of recently reported state-of-the-art catalysts for EGOR [^1-18^](#_ENREF_1).





**Supplementary Fig. 8 FTIR spectra of Pd/NiMoO_4_/NF and NiMoO_4_/NF.**


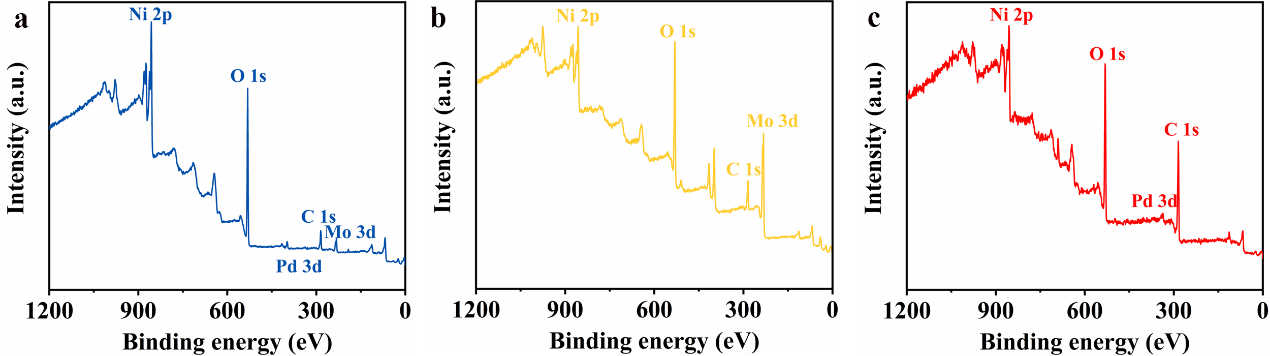


**Supplementary Fig. 9 XPS spectra.** XPS survey spectrum of (a) Pd/NiMoO_4_/NF, (b) NiMoO_4_/NF, (c) Pd/NF.


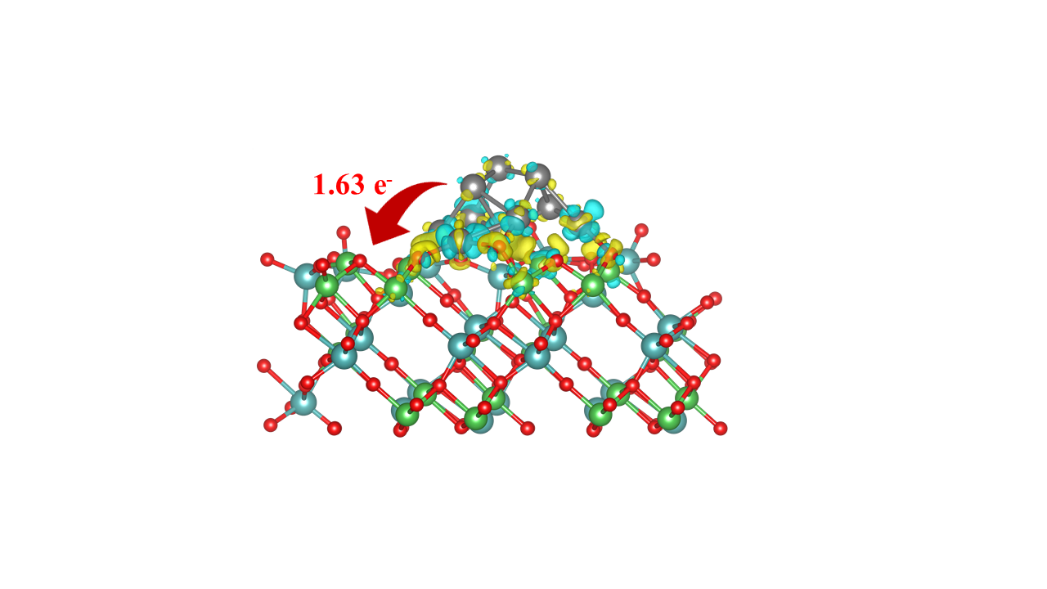


**Supplementary Fig. 10 Charge density difference.** Where the blue and yellow isosurfaces denote the electron depletion and accumulation on Pd/NiMoO_4_.





**Supplementary Fig. 11 D-Band center analysis.** XPS valence band spectra of NiMoO_4_/NF.





**Supplementary Fig. 12 The DFT results of d-Band cente.** PDOS (d-band) of Pd and Pd/NiMoO_4_.





**Supplementary Fig. 13 Electrochemical test.** LSV curves of Pd/NiMoO_4_/NF anode in 1.0 M NaOH with or without 1 M ethylene glycol addition. Scan rate, 10 mV s^−1^.





**Supplementary Fig. 14 The Comparisons of the potentials.** Comparisons of the potentials needed to achieve designated current densities for Pd/NiMoO_4_/NF in 1.0 M NaOH with and without EG.


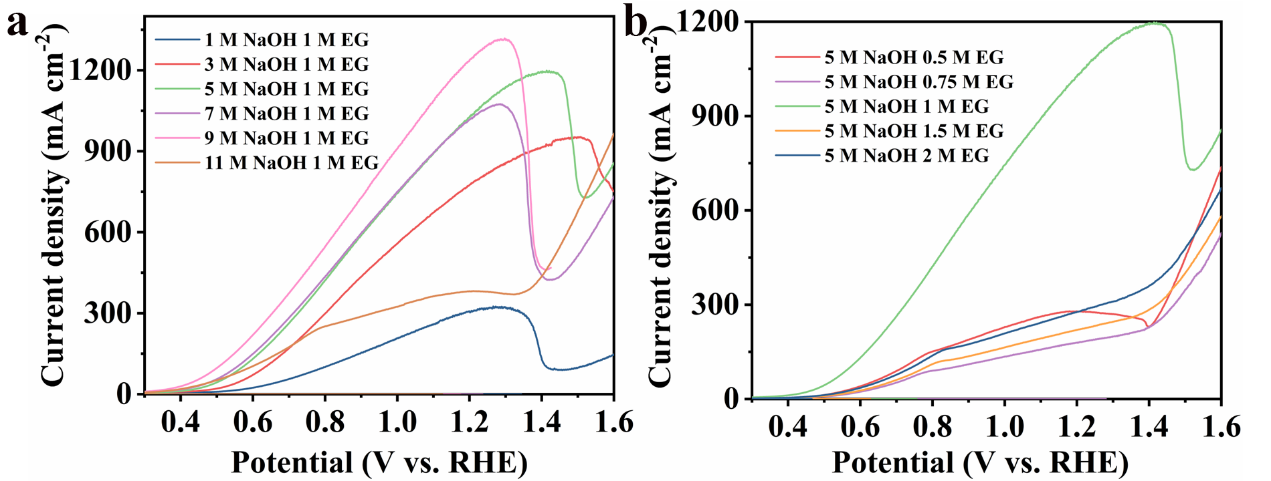


**Supplementary Fig. 15 Electrochemical tests.** LSV curves of Pd/NiMoO_4_/NF at scan rate of 10 mV s^−1^ in (a)1 M EG solution with different concentrations of NaOH and (b) in 5 M NaOH with varied ethylene glycol concentrations.

**Supplementary Note 6**

Control samples have been synthesized and corresponding experiments have been carried out. The control precursors were prepared by a similar procedure with varied Na_2_PdCl_4_ concentration. The precise Pd loading amount has been analyzed by inductively coupled plasma mission spectroscopy (ICP-OES)，and the weight percentage of Pd is 1.7% and 6.9% for Pd_1.7%_/NiMoO_4_/NF and Pd_6.9%_/NiMoO_4_/NF. As shown in **Supplementary Figure 16a**, the XRD patterns indicates the successful synthesis of the Pd_1.7%_/NiMoO_4_/NF and Pd_6.9%_/NiMoO_4_/NF controlled samples. Noticeably, Pd/NiMoO_4_/NF with 3.5 wt.% Pd content shows significantly higher current density than Pd_1.7%_/NiMoO_4_/NF and Pd_6.9%_/NiMoO_4_/NF for electrocatalytic ethylene glycol oxidation (**Supplementary Figure 16b**). Furthermore, the reason for the much enhanced electrocatalytic activity has been investigated by measuring the adsorption of the OH^-^ and EG species of these electrocatalysts. As shown in the CV curves (**Figure 5a** and **Supplementary Figure 17a**), Pd/NiMoO_4_/NF exhibits stronger OH^-^ adsorption bands at significantly lower onset potential (0.5 V vs. RHE and 1.0 vs. RHE) than Pd_1.7%_/NiMoO_4_/NF and Pd_6.9%_/NiMoO_4_/NF Besides, as shown in **Supplementary Figure 17b,** upon adding 1 M EG, the OCP is significantly decreased for Pd/NiMoO_4_/NF (Δ = 0.68 V) compared with Pd_1.7%_/NiMoO_4_/NF (Δ = 0.52 V) and Pd_6.9%_/NiMoO_4_/NF (Δ = 0.65 V), indicating the much enhanced EG adsorption of Pd/NiMoO_4_/NF. The above results indicate that the moderate Pd content is contributed to the adsorption of OH^-^ and EG and thus enhance the oxidation performance of EG.


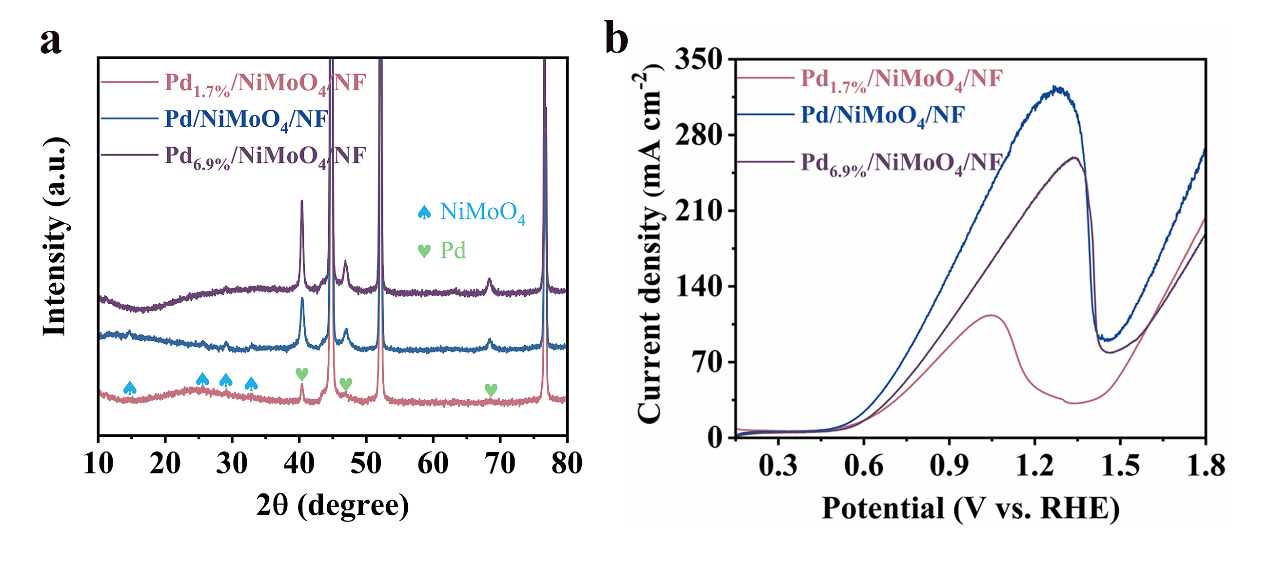


**Supplementary Fig. 16 The structure of the contrast samples and the corresponding electrochemical tests.** **a** XRD patterns of Pd_1.7%_/NiMoO_4_/NF, Pd_3.5%_/NiMoO_4_/NF and Pd_6.9%_/NiMoO_4_/NF. **b** LSV curves of obtained electrocatalysts for ethylene glycol anodic oxidation.


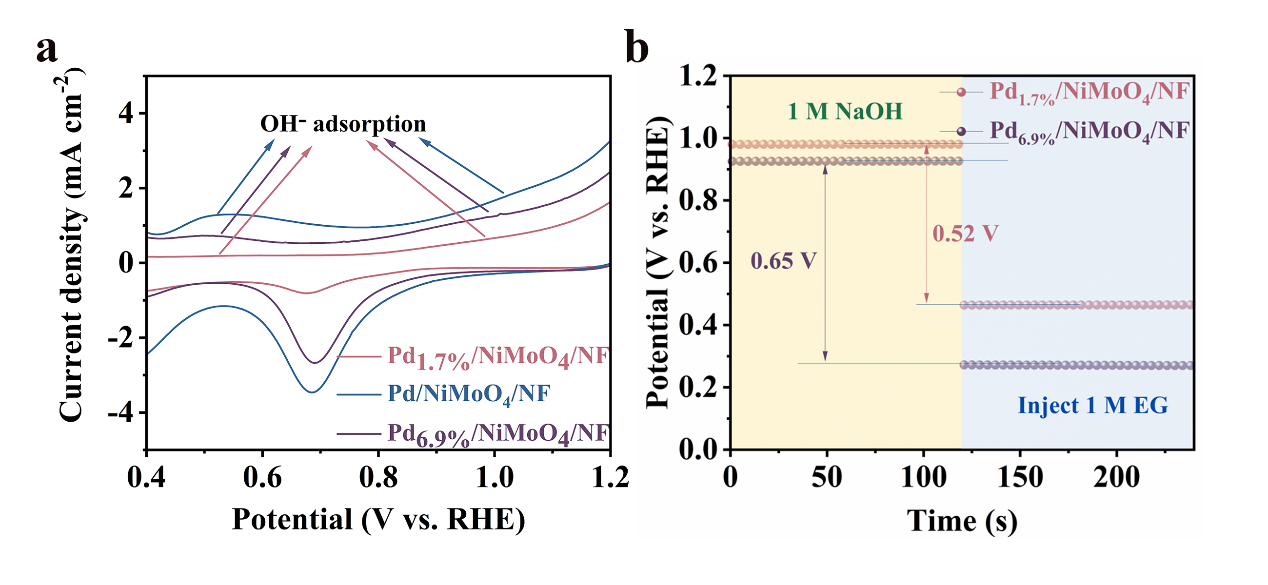


**Supplementary Fig. 17 CV and OCPs tests.** **a** CV curves of Pd/NiMoO_4_/NF, Pd_1.7%_/NiMoO_4_/NF and Pd_3.5%_/NiMoO_4_/NF in 1 M NaOH. **b** OCPs of Pd_1.7%_/NiMoO_4_/NF and Pd_3.5%_/NiMoO_4_/NF in 1 M NaOH solution with or without EG addition.

**Supplementary Note 7**

Control samples NiO/NF, Pd/NiO/NF, MoO_3_/NF and Pd/MoO_3_/NF have been synthesized and corresponding experiments have been carried out. The control precursors were prepared by a similar procedure but without the addition of Ni(NO_3_)_2_·6H_2_O or Na_2_MoO_4_·2H_2_O, which were annealed in Ar atmosphere at the temperature of 450 °C for 2 h in a tubular furnace to obtain the contrast samples. As shown in **Supplementary Figure 18a**, the XRD patterns of these materials indicate the successful formation of the NiO/NF, Pd/NiO/NF, MoO_3_/NF and Pd/MoO_3_/NF. Noticeably, the peak current density of Pd/NiMoO_4_/NF is significantly higher than those of Pd/NiO/NF and Pd/MoO_3_/NF for electrocatalytic ethylene glycol oxidation (**Supplementary Figure 18b**). Furthermore, the adsorption of the OH^-^ and EG species were determined by the CV and OCP measurements. As shown in **Figure 5a** and **Supplementary Figure 19a**, Pd/NiMoO_4_/NF exhibits OH^-^ adsorption bands at significantly lower onset potential (0.5 V vs RHE) than Pd/NiO/NF (0.65 V vs RHE) and Pd/MoO_3_/NF (no obvious adsorption bands). Besides, as shown in **Supplementary Figure 19b,** upon adding 1 M EG, the OCP of Pd/NiMoO_4_/NF is significantly decreased (Δ = 0.68 V) compared with Pd/NiO/NF (Δ = 0.65 V) and Pd/MoO_3_/NF (Δ = 0.49 V). The above results indicate that the choice of NiMoO_4_ as the support for the deposition of Pd is contributed to the adsorption of OH^-^ and EG and thus enhance the oxidation performance of EG.


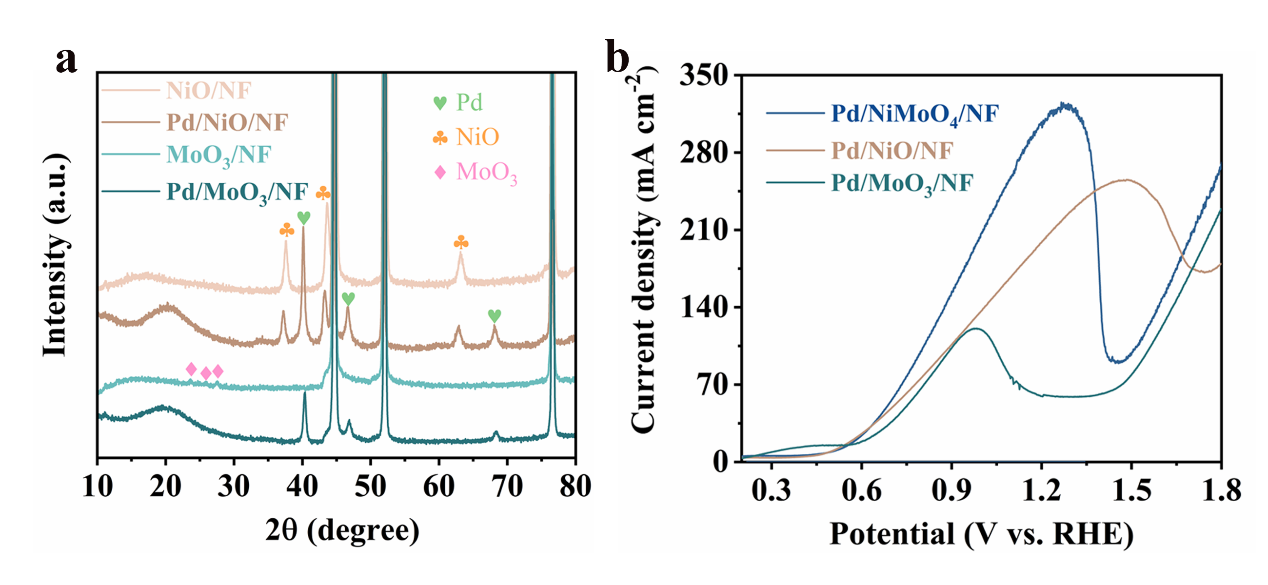


**Supplementary Fig. 18** **The structure of the contrast samples and the corresponding electrochemical tests.** **a** XRD patterns of NiO/NF, Pd/NiO/NF, MoO_3_/NF and Pd/MoO_3_/NF. **b** LSV curves of obtained electrocatalysts for ethylene glycol anodic oxidation.


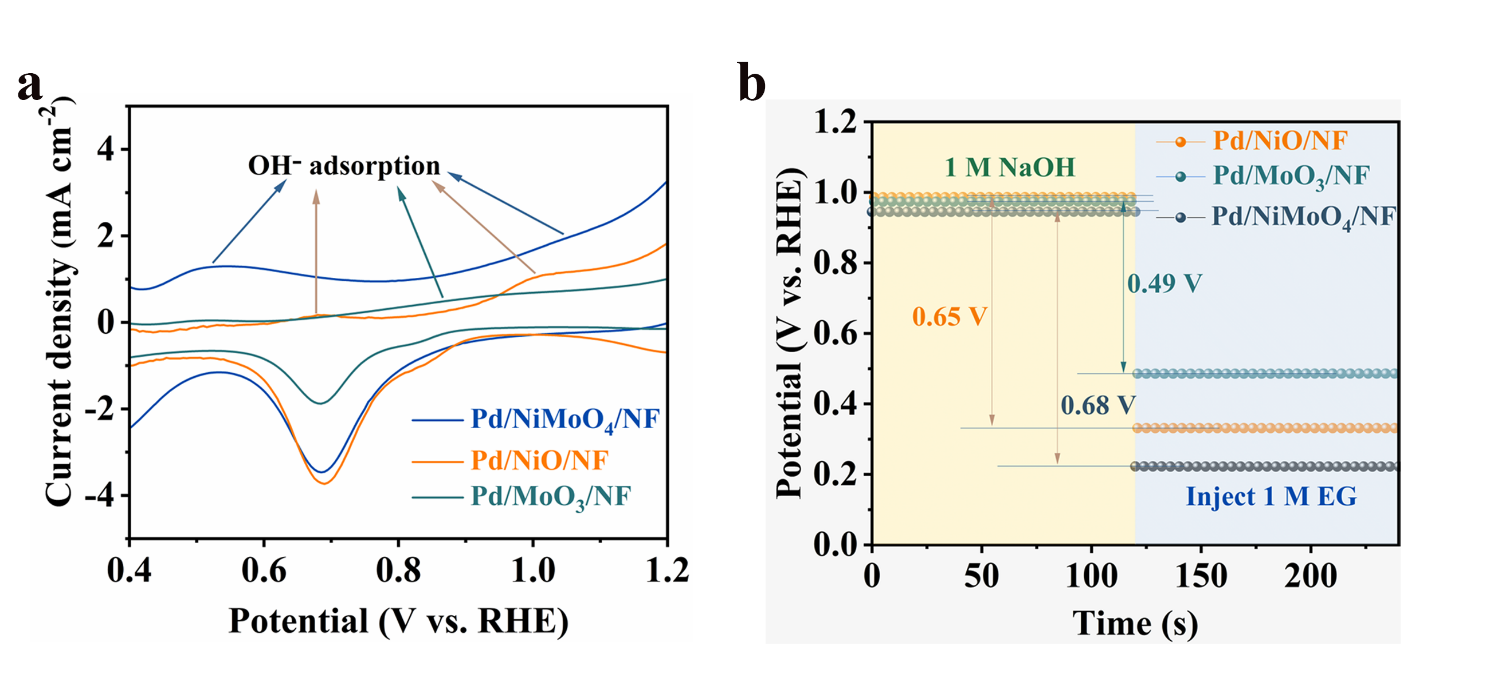


**Supplementary Fig. 19** **CV and OCPs tests.** **a** CV curves of Pd/NiMoO_4_/NF, Pd/NiO/NF and Pd/MoO_3_/NF in 1 M NaOH. **b** OCPs of Pd/NiO/NF and Pd/MoO_3_/NF in 1 M NaOH solution with or without EG addition.


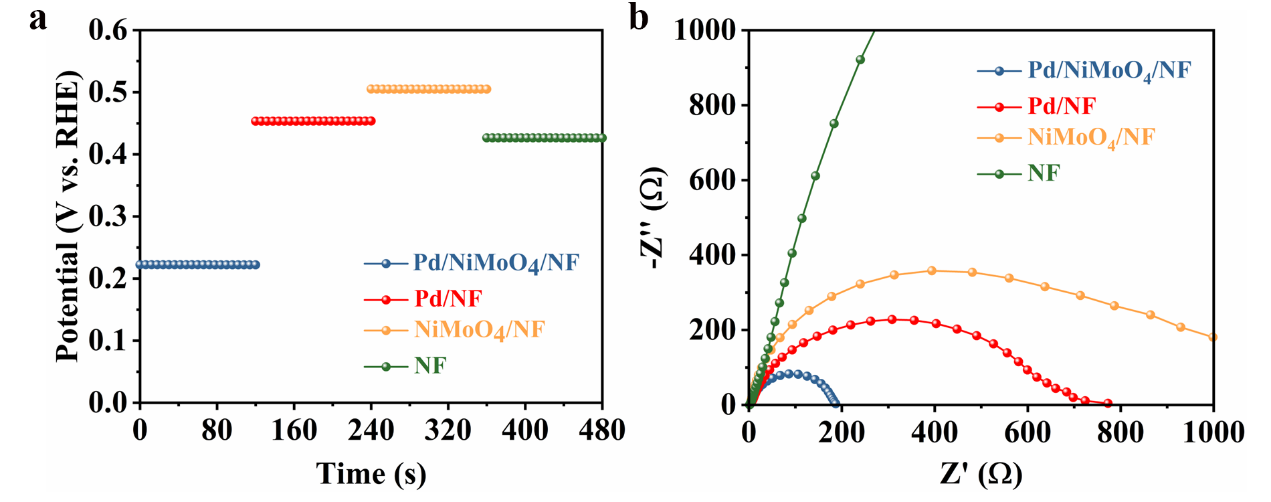


**Supplementary Fig. 20** **OCPs and EIS tests. a** OCP and **b** EIS plots of Pd/NiMoO_4_/NF, NiMoO_4_/NF, Pd/NF, and pure NF.


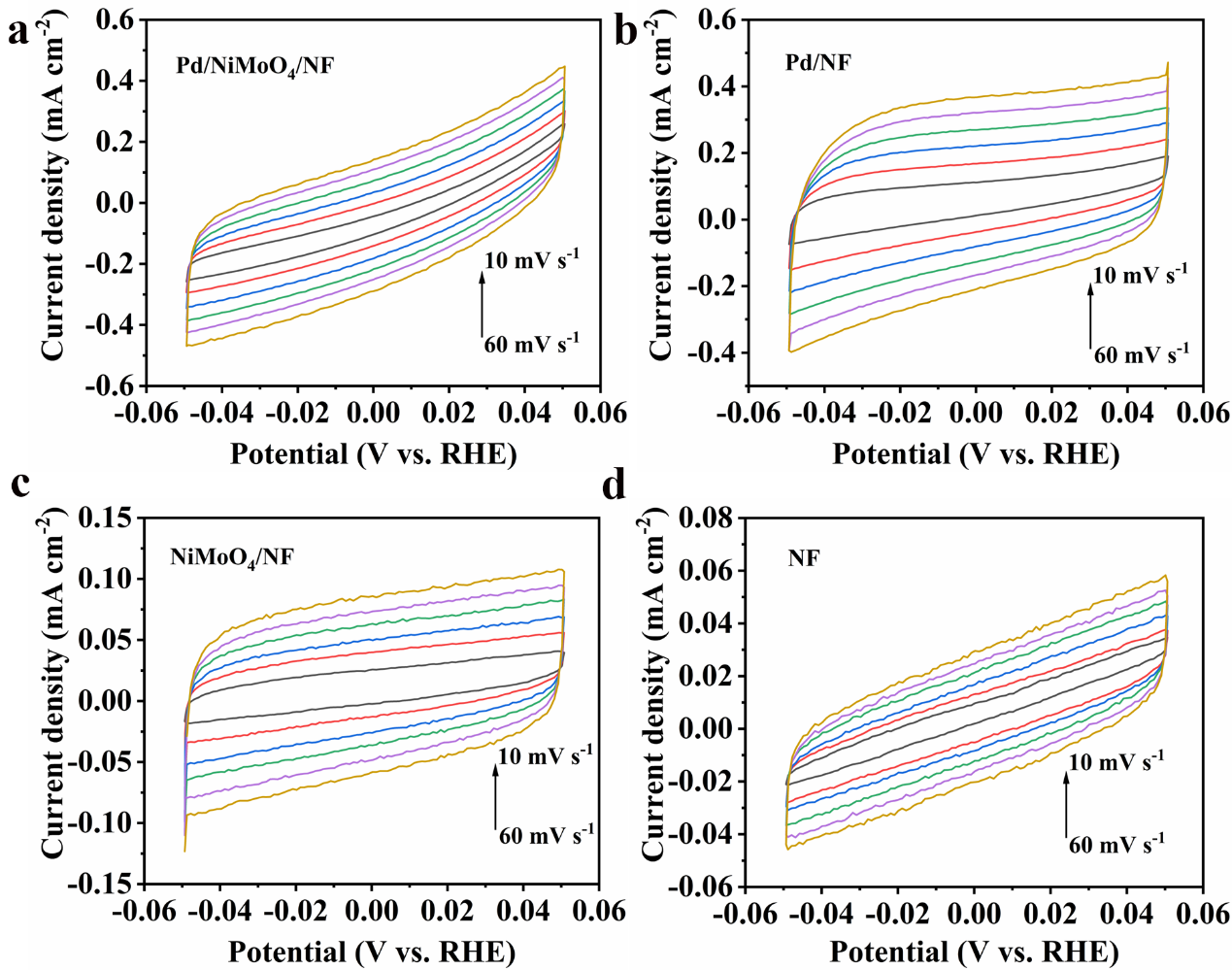


**Supplementary Fig. 21** **CV curves with different rate from 20 to 100 mV s^-1^.**CV curves of **a** Pd/NiMoO_4_/NF, **b** Pd/NF, **c** NiMoO_4_/NF and **d** pure NF in the non-faradic current range under different scan rates (10-60 mV/s) in 1.0 M NaOH with 1M EG.





**Supplementary Fig. 22** **ECSA-normalized LSV curves.** ECSA-normalized LSVs of Pd/NiMoO_4_/NF, NiMoO_4_/NF, Pd/NF, and pure NF.





**Supplementary Fig. 23 ^1^H NMR spectra.** ^1^H NMR spectra of the electrolyte after 2 h anodic ethylene glycol oxidation on a Pd/NiMoO_4_/NF electrode using maleic acid as the internal standard.

**Supplementary Note 8**

The ^13^C NMR spectra of the product has been provided in the revised supporting information. As shown in **Supplementary Figure 24**, the ^13^C NMR spectrum of the electrolyte after 2 h anodic ethylene glycol oxidation on Pd/NiMoO_4_/NF electrode shows obvious peaks at approximately 61.3 ppm and 180.2 ppm, which is identical with that of sodium glycolate, indicating the main oxidation product is sodium glycolate (62.7 ppm refer to the ethylene glycol).





**Supplementary Fig. 24** **^13^C NMR spectra.** ^13^C NMR spectra of the electrolyte before and after 2 h anodic ethylene glycol oxidation on Pd/NiMoO_4_/NF electrode.





**Supplementary Fig. 25 ^1^H NMR spectra.** ^1^H NMR spectra of the electrolyte after 2 h anodic ethylene glycol oxidation on Pd/NiMoO_4_/NF electrode at varied potentials.





**Supplementary Fig. 26 The total charges in different cycles.** The total charges of Pd/NiMoO_4_/NF for sodium glycolate production for 62 electrolysis cycles.





**Supplementary Fig. 27 The Comparisons of the stability.** A comparison of the performed catalytic durations among recently reported state-of-the-art catalysts for EGOR.





**Supplementary Fig. 28 I-t tests.** FEs and current density of Pd /NF for sodium glycolate production for 72 h electrolysis cycles.


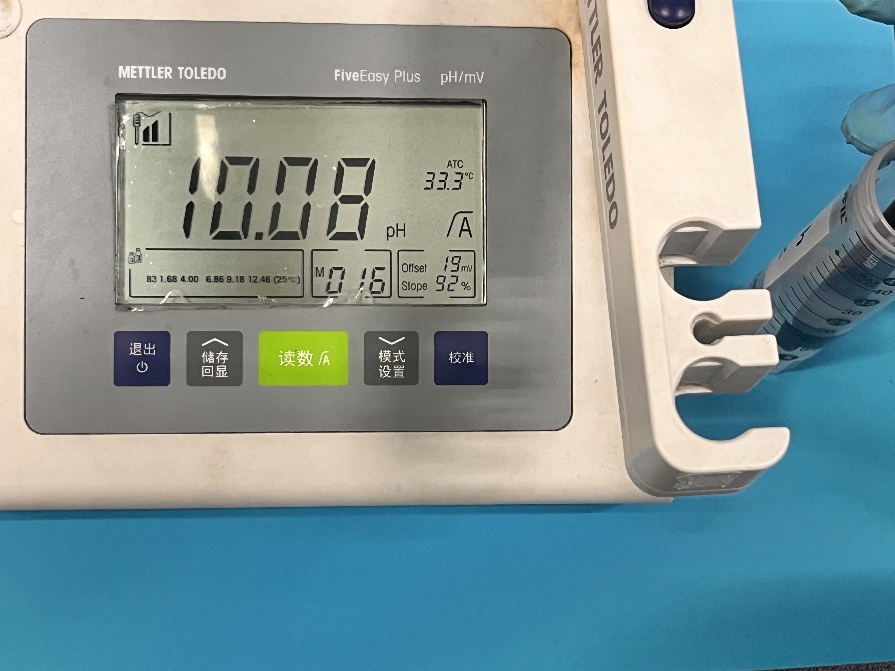


**Supplementary Fig. 29 The photo of pH.** The pH of the electrolyte after EGOR.


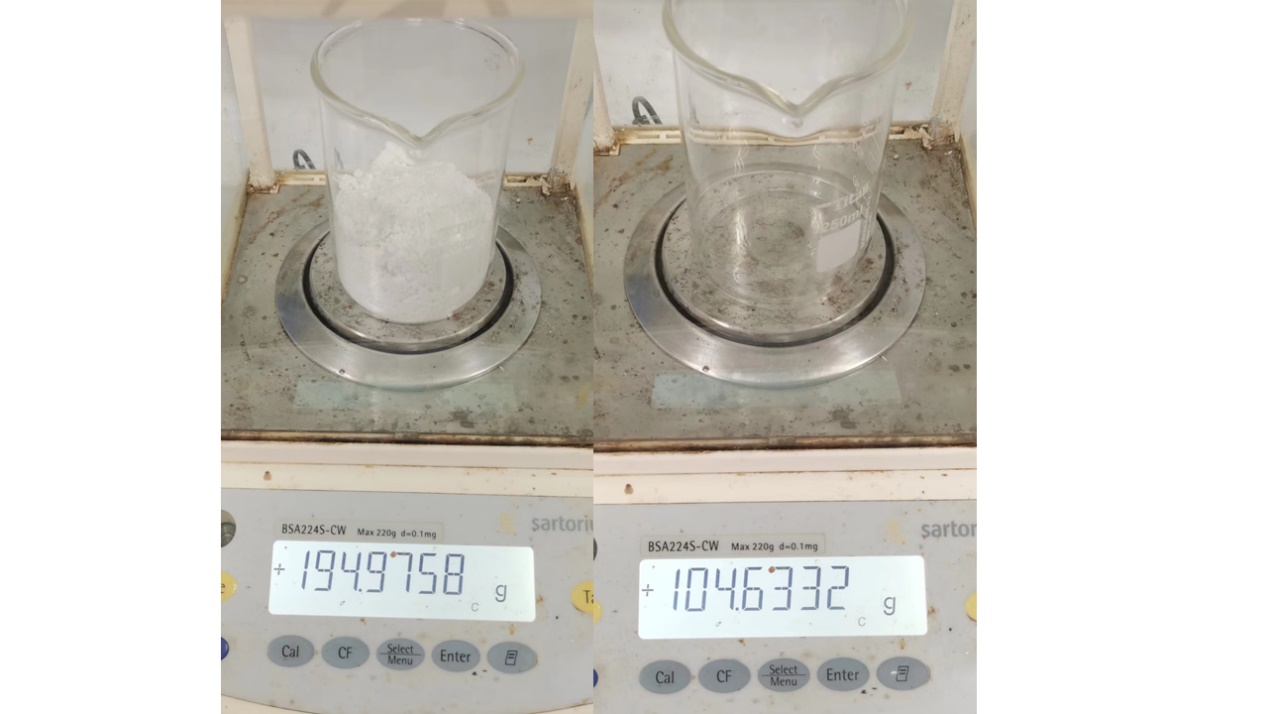


**Supplementary Fig. 30 The photo of products mass.** The photo of prepared products.





**Supplementary Fig. 31** **XRD patterns.** XRD patterns of Pd/NiMoO_4_/NF electrode before and after electrolysis.


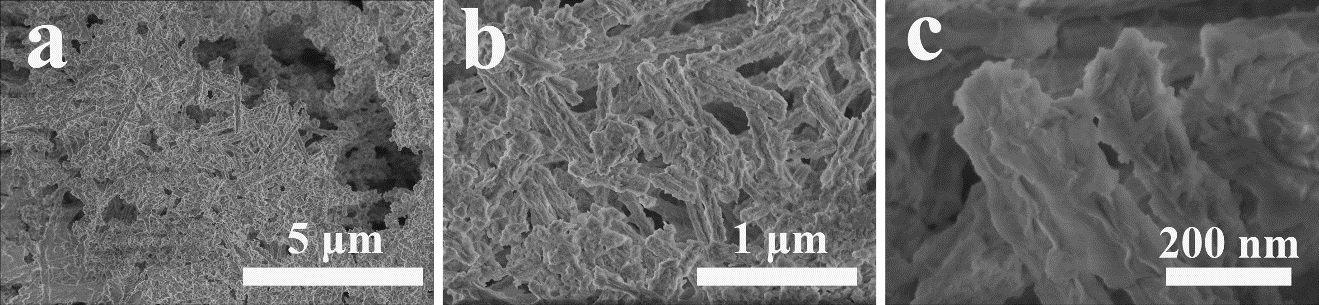


**Supplementary Fig. 32** **SEM images of Pd/NiMoO_4_ with different scales.** SEM images of Pd/NiMoO_4_/NF electrode after electrolysis **a** 5 μm, **b** 1μm, **c** 200 nm.


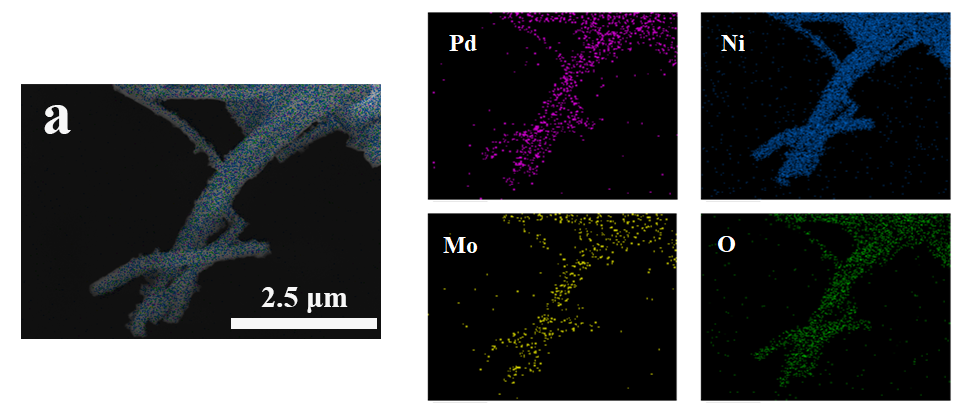


**Supplementary Fig. 33** **EDS spectrum.** EDS spectrum of Pd/NiMoO_4_/NF electrode after electrolysis.

**
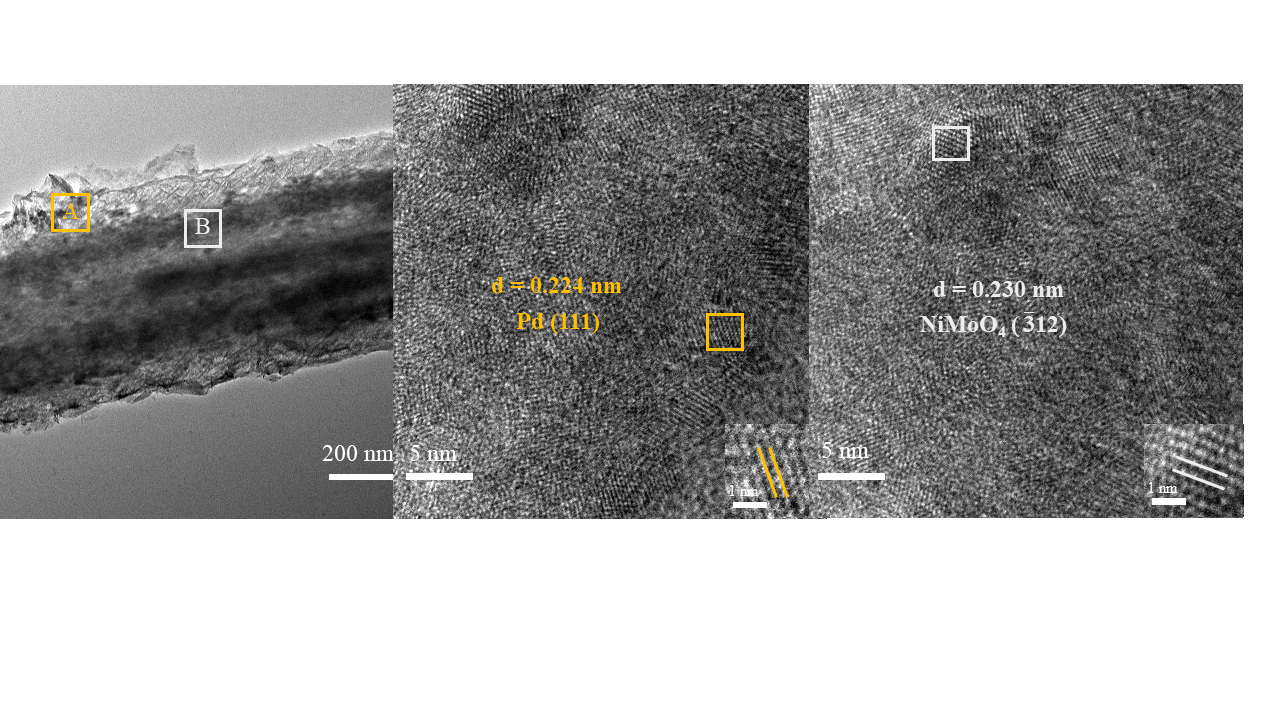
**

**Supplementary Fig. 34** **HRTEM images.** HRTEM image of Pd/NiMoO_4_/NF electrode after electrolysis.





**Supplementary Fig. 35** **Standard curve.** The standard curve of H_2_ production performed on gas chromatography (argon as a carrier gas).





**Supplementary Fig. 36** **The Faradic efficiency of H_2_.** Faradic efficiency of HER in a three-electrode system at varied charges.





**Supplementary Fig. 37** **OCPs test.** OCPs of NiMoO_4_/NF in 1 M NaOH solution before and after EG was added.


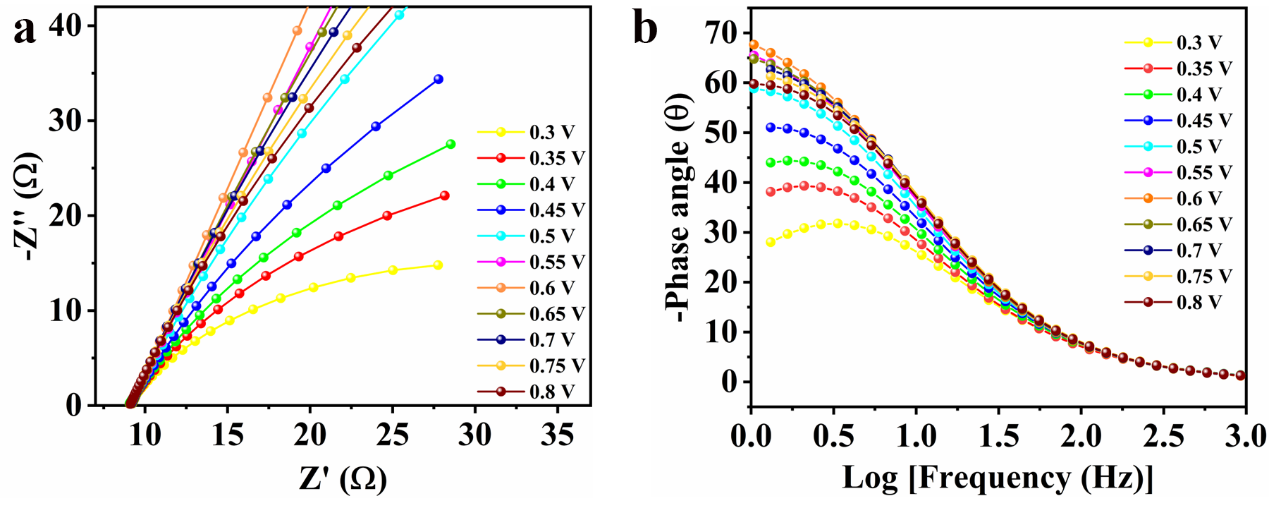


**Supplementary Fig. 38 In-situ EIS tests.** The Nyquist plots **a** and **b** corresponding Bode phase plots of Pd/NiMoO_4_/NF electrode at varied potentials in 1 M NaOH with 1 M GA.





**Supplementary Fig. 39** **LSV curves.** LSV curves of Pd/NiMoO_4_/NF in 1 M NaOH with 1 M EG or1 M GA.





**Supplementary Fig. 40 CO stripping experiments of of Pd/NF.**





**Supplementary Fig. 41 CO stripping experiments of NiMoO_4_/NF.**





**Supplementary Fig. 42** **In-situ FTIR spectra.** In-situ electrochemical FTIR spectra of EGOR catalyzed by Pd/NF.

^
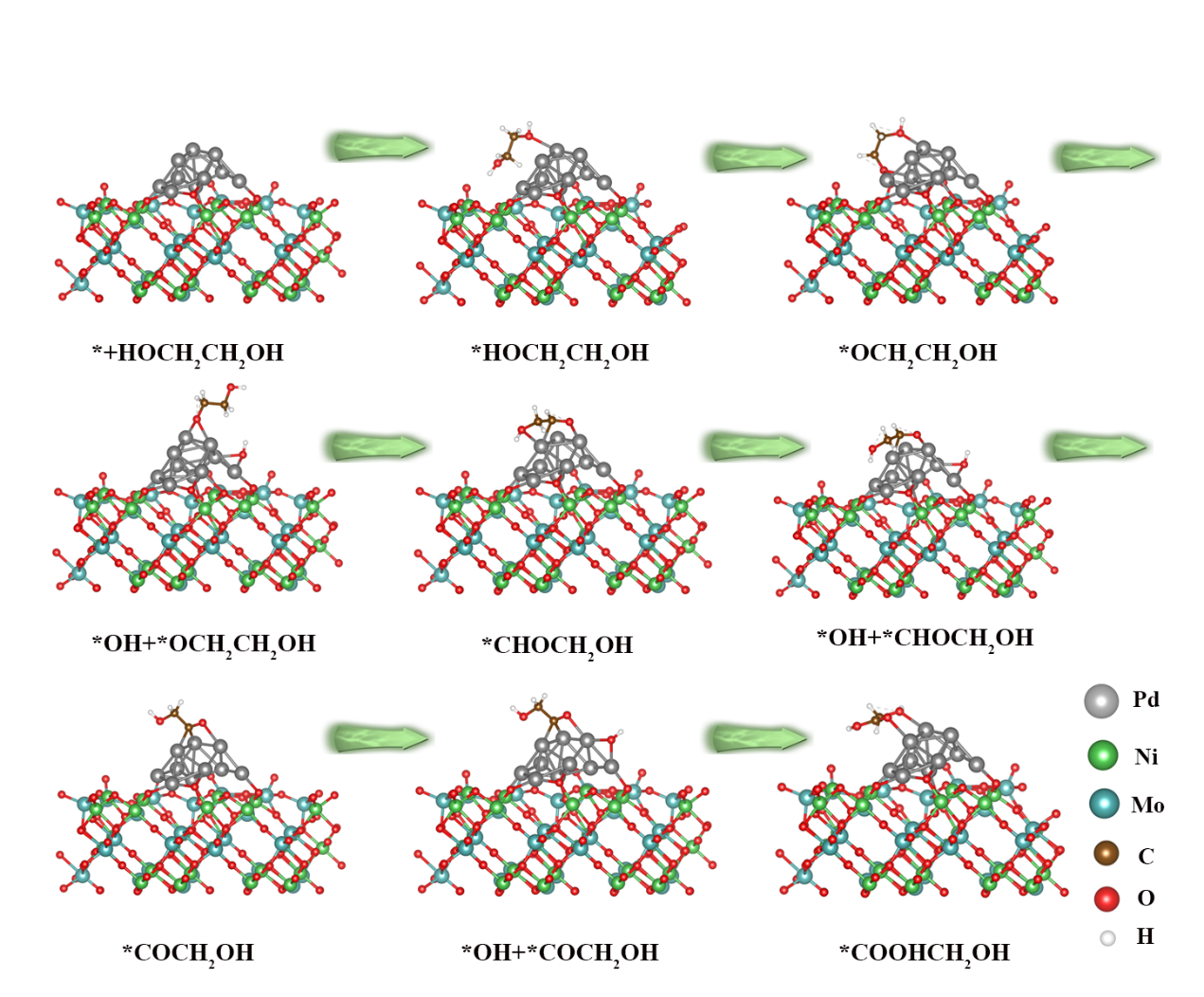
^

**Supplementary Fig. 43** **Optimum adsorption model.** The optimized models of the EG oxidation on the Pd/NiMoO_4_ catalysts.


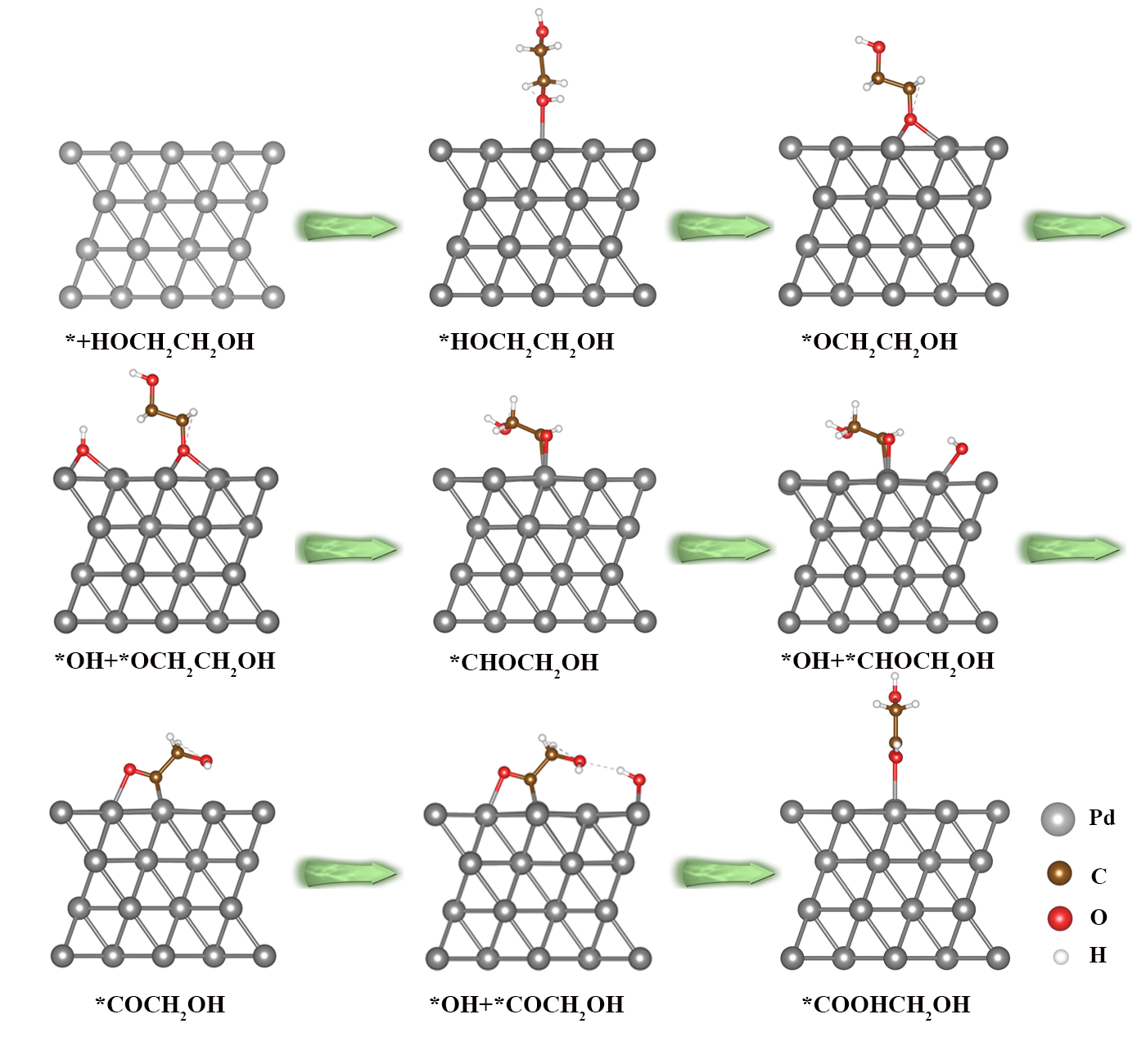


**Supplementary Fig. 44** **Optimum adsorption model.** The optimized models of the EG oxidation on the Pd catalysts.





**Supplementary Fig. 45** **Gibbs free energy landscapes.** Gibbs free energy landscapes for EGOR to glycolic acid over Pd/NiMoO_4_ and Pd at 0 V vs RHE.


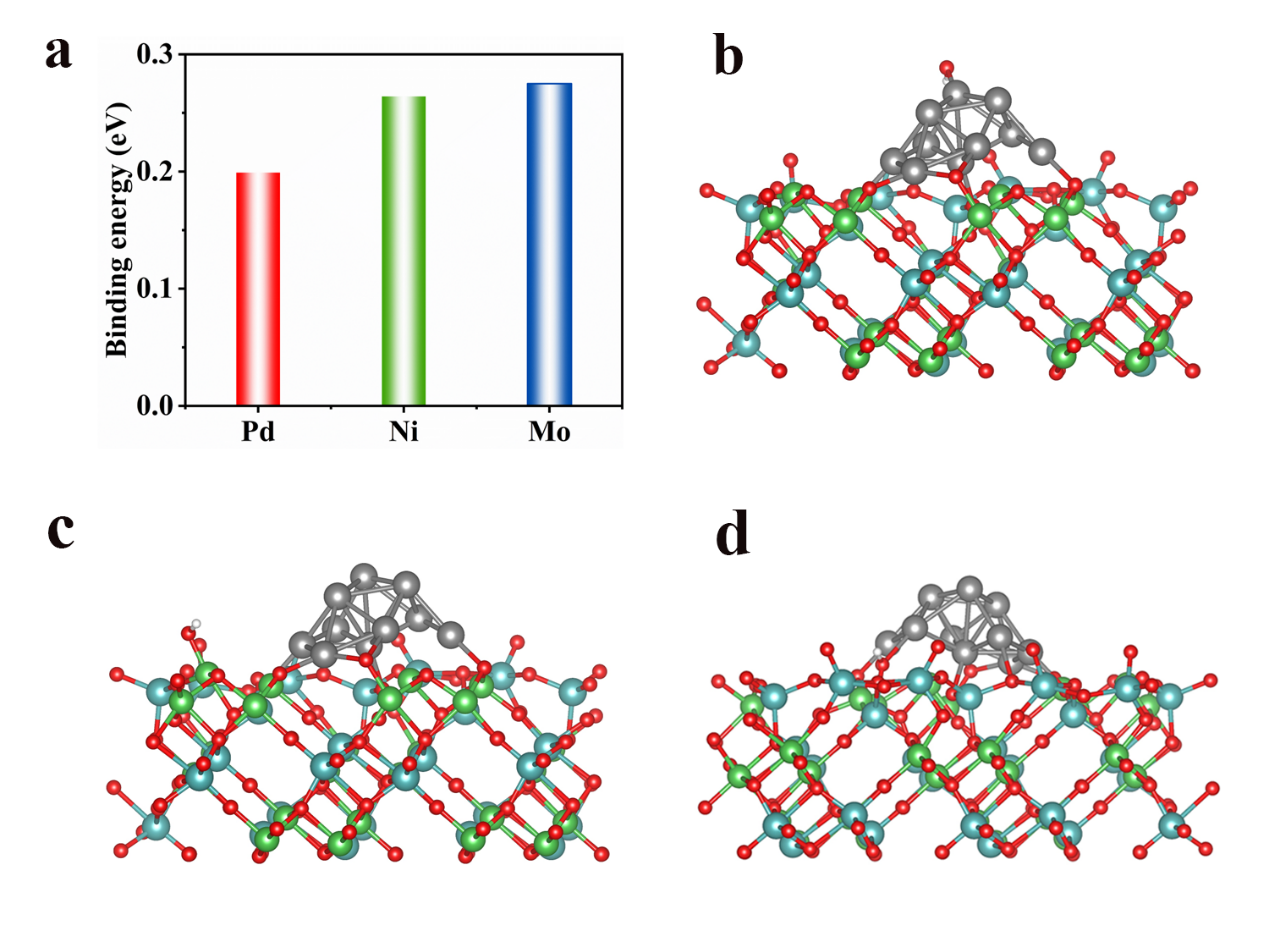


**Supplementary Fig. 46** **Calculated adsorption energies and adsorption model. a** Calculated adsorption energies of OH on the surfaces of Pd/NiMoO_4_ at different adsorption sites. **b** Pd adsorption site. **c** Ni adsorption site. **d** Mo adsorption site.





**Supplementary Fig. 47 Calculated adsorption energies.** Calculated adsorption energies of EG on the surfaces of Pd (111) and Pd/NiMoO_4_.


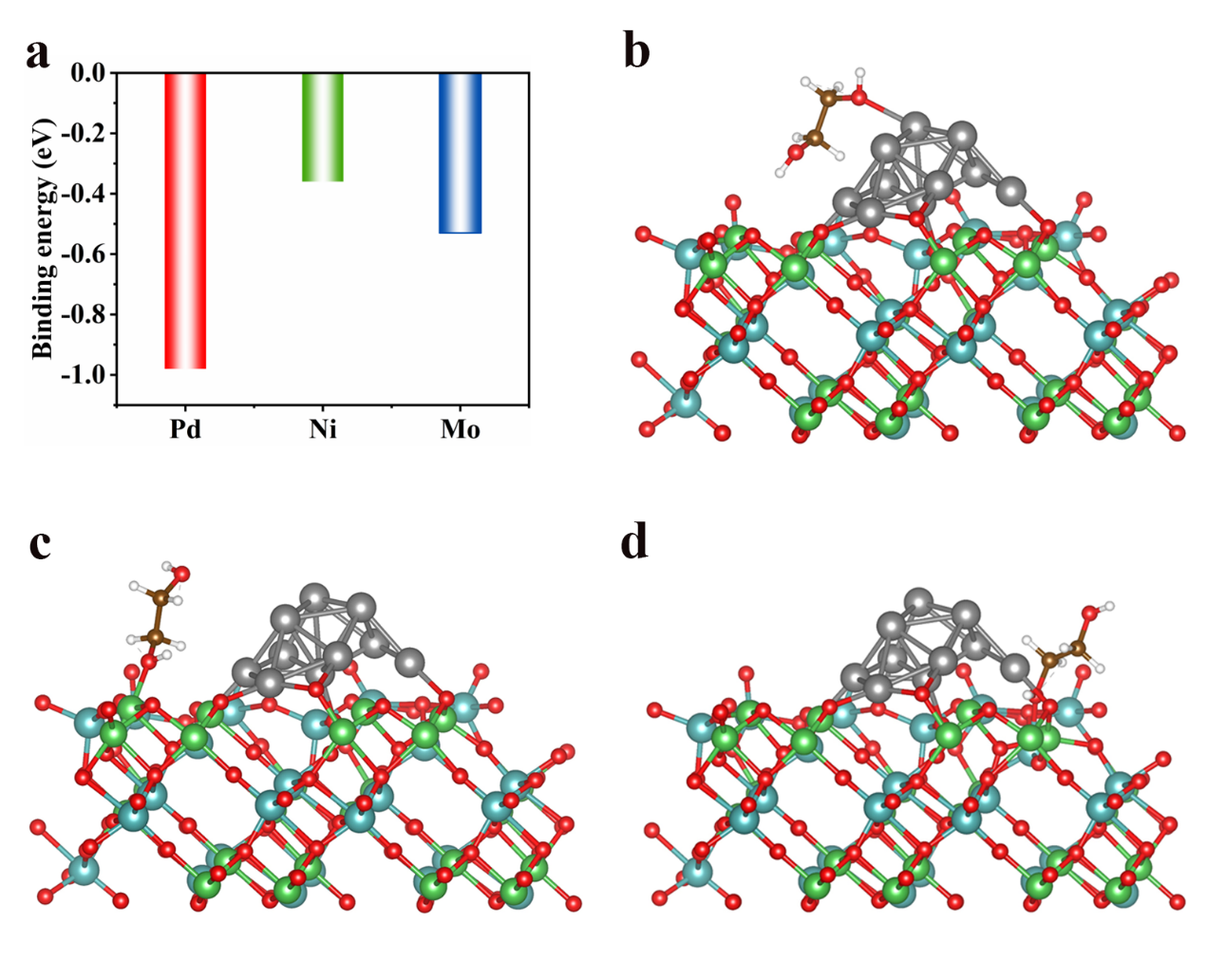


**Supplementary Fig. 48 Calculated adsorption energies and adsorption model.** **a** Calculated adsorption energies of EG on the surfaces of Pd/NiMoO_4_ at different adsorption sites. **b** Pd adsorption site. **c** Ni adsorption site. **d** Mo adsorption site.


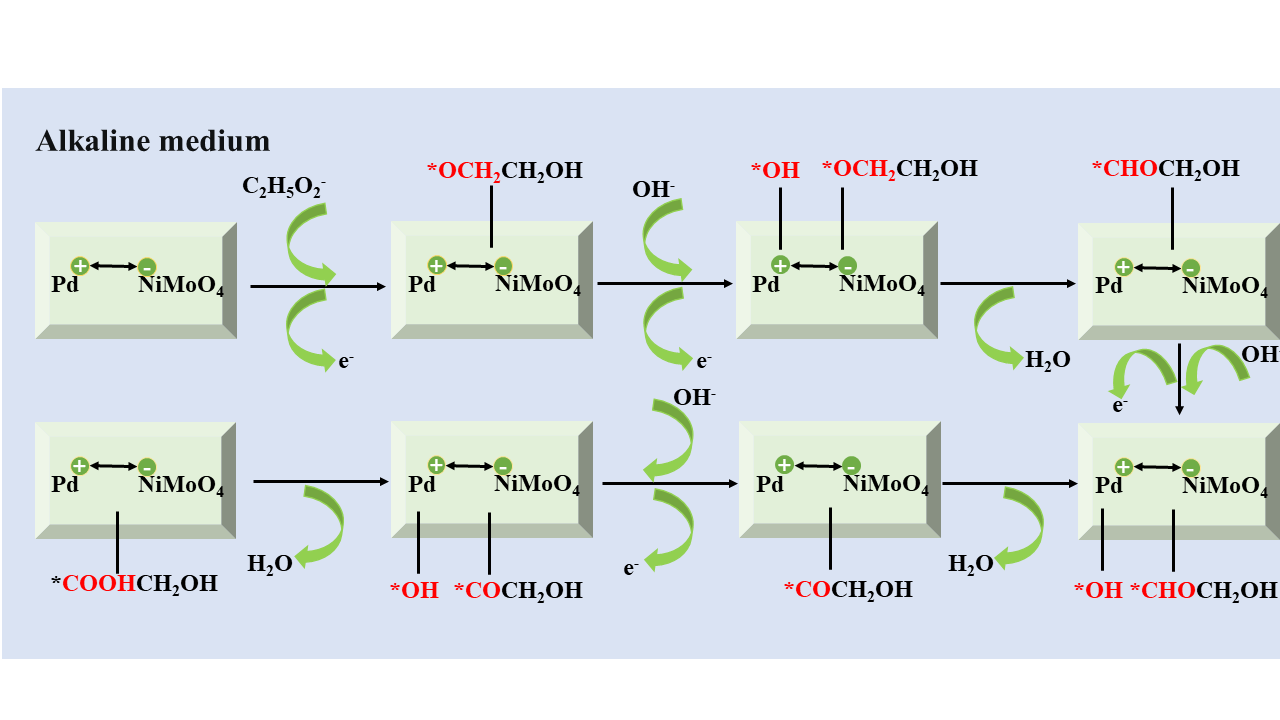


**Supplementary Fig. 49 Possible reaction path.** Proposed dominant pathway of EG electrocatalytic oxidation to GA on Pd/NiMoO_4_/NF in an alkaline medium.

**Supplementary Note 9 for DFT calculation**

We have employed the Vienna Ab Initio Package (VASP)^19,20^ to perform all the density functional theory (DFT) calculations within the generalized gradient approximation (GGA) using the PBE^21^ formulation. We have chosen the projected augmented wave (PAW) potentials^22,23^ to describe the ionic cores and take valence electrons into account using a plane wave basis set with a kinetic energy cutoff of 400 eV. Partial occupancies of the Kohn−Sham orbitals were allowed using the Gaussian smearing method and a width of 0.05 eV. The electronic energy was considered self-consistent when the energy change was smaller than 10^−5^ eV. A geometry optimization was considered convergent when the force change was smaller than 0.02 eV/Å. Grimme’s DFT-D3 methodology^24^ was used to describe the dispersion interactions.

The equilibrium lattice constant of FCC Pd unit cell was optimized to be a=3.886 Å. We then used it to construct a Pd (111) surface model (model 1) with p (3×2$\sqrt{3}$) periodicity in the x and y directions and 4 atomic layers in the z direction separated by a vacuum layer in the depth of 15 Å in order to separate the surface slab from its periodic duplicates. Model 1 comprises of 48 Pd atoms. During structural optimizations, a 2×2×1 k-point grid in the Brillouin zone was used for k-point sampling, and the bottom two atomic layers were fixed while the top two were allowed to relax.

The equilibrium lattice constants of monoclinic NiMoO_4_ unit cell were optimized to be a=9.468 Å, b=8.650 Å, c=7.576 Å, α=90°, β=114.2°, γ=90°. We then use it to construct a NiMoO_4_ ($\bar{\text{3}}\text{12}$) surface model with p (2×1) periodicity in the x and y directions and 3 stoichiometric layers in the z direction separated by a vacuum layer in the depth of 15 Å in order to separate the surface slab from its periodic duplicates. Model 2 was built by one Pd10 cluster residing onto this NiMoO_4_ ($\bar{\text{3}}\text{12}$) surface. Model 2 comprises of 10 Pd, 24 Ni, 24 Mo and 96 O atoms. During structural optimizations, a 1×2×1 k-point grid in the Brillouin zone was used for k-point sampling, and the bottom two stoichiometric layers were fixed while the rest were allowed to relax.

The adsorption energy (E_ads_) of adsorbate A was defined as:

E_ads_ = E_A/surf_ – E_surf_ – E_A(g)_

where E_A/surf_, E_surf_ and E_A(g)_ are the energy of adsorbate A adsorbed on the surface, the energy of clean surface, and the energy of isolated A molecule in a cubic periodic box with a side length of 20 Å and a 1×1×1 Monkhorst-Pack k-point grid for Brillouin zone sampling, respectively.

The free energy of a gas phase molecule or an adsorbate on the surface was calculated by the equation G = E + ZPE − TS, where E is the total energy, ZPE is the zero-point energy, T is the temperature in kelvin (298.15 K is set here), and S is the entropy.

**Supplementary References**

1. Liu, F.; Gao, X.; Shi, R.; Guo, Z.; Tse, E. C. M., et al., Concerted and Selective Electrooxidation of Polyethylene-Terephthalate-Derived Alcohol to Glycolic Acid at an Industry-Level Current Density over a Pd-Ni(OH)_2_ Catalyst. *Angew. Chem. Int. Ed.* **62,** e202300094 (2023).

2. Yan, Y.; Zhou, H.; Xu, S. M.; Yang, J.; Hao, P., et al., Electrocatalytic Upcycling of Biomass and Plastic Wastes to Biodegradable Polymer Monomers and Hydrogen Fuel at High Current Densities. *J. Am. Chem. Soc.* **145,** 6144-6155 (2023).

3. Arjona, N.; Espinosa‐Magaña, F.; Bañuelos, J. A.; Álvarez‐Contreras, L.; Guerra‐Balcázar, M., Manganese Oxides (Mn_3_O_4_&α‐MnO_2_) as Co‐catalysts in Pd‐Based Nanomaterials for the Ethylene Glycol Electro‐Oxidation. *ChemElectroChem* **9,** 202200015 (2022).

4. Bai, S.; Xu, Y.; Cao, K.; Huang, X., Selective Ethanol Oxidation Reaction at the Rh-SnO_2_ Interface. *Adv. Mater.* **33,** e2005767 (2021).

5. Guo, X.; Shang, H.; Guo, J.; Xu, H.; Du, Y., Ultrafine two-dimensional alloyed PdCu nanosheets-constructed three-dimensional nanoflowers enable efficient ethylene glycol electrooxidation. *Appl. Surf. Sci.* **481,** 1532-1537 (2019).

6. Zhu, J.; Xia, L.; Yu, R.; Lu, R.; Li, J., et al., Ultrahigh Stable Methanol Oxidation Enabled by a High Hydroxyl Concentration on Pt Clusters/MXene Interfaces. *J. Am. Chem. Soc.* **144,** 15529-15538 (2022).

7. Fan, L.; Ji, Y.; Wang, G.; Chen, J.; Chen, K., et al., High Entropy Alloy Electrocatalytic Electrode toward Alkaline Glycerol Valorization Coupling with Acidic Hydrogen Production. *J. Am. Chem. Soc.* **144,** 7224-7235 (2022).

8. Yang, X.; Yao, K. x.; Ye, J. Y.; Yuan, Q.; Zhao, F., et al., Interface‐Rich Three‐Dimensional Au‐Doped PtBi Intermetallics as Highly Effective Anode Catalysts for Application in Alkaline Ethylene Glycol Fuel Cells. *Adv. Funct. Mater.* **31,** 2103671 (2021).

9. Zhang, S.; Liu, K.; Liu, Z.; Liu, M.; Zhang, Z., et al., Highly Strained Au-Ag-Pd Alloy Nanowires for Boosted Electrooxidation of Biomass-Derived Alcohols. *Nano Lett.* **21,** 1074-1082 (2021).

10. Qin, Y.; Zhang, W.; Wang, F.; Li, J.; Ye, J., et al., Extraordinary p-d Hybridization Interaction in Heterostructural Pd-PdSe Nanosheets Boosts C-C Bond Cleavage of Ethylene Glycol Electrooxidation. *Angew. Chem. Int. Ed.* **61,** e202200899 (2022).

11. Li, Z.-Y.; Zhou, J.; Tang, L.-S.; Fu, X.-P.; Wei, H., et al., Hydroxyl-rich ceria hydrate nanoparticles enhancing the alcohol electrooxidation performance of Pt catalysts. *J. Mater. Chem. A* **6,** 2318-2326 (2018).

12. Chen, Y.; Zheng, X.-X.; Huang, X.-Y.; Wang, A.-J.; Zhang, Q.-L., et al., Trimetallic PtRhCo petal-assembled alloyed nanoflowers as efficient and stable bifunctional electrocatalyst for ethylene glycol oxidation and hydrogen evolution reactions. *J Colloid Interf Sci* **559,** 206-214 (2020).

13. Wang, Y.; Zhuo, H.; Sun, H.; Zhang, X.; Dai, X., et al., Implanting Mo Atoms into Surface Lattice of Pt_3_Mn Alloys Enclosed by High-Indexed Facets: Promoting Highly Active Sites for Ethylene Glycol Oxidation. *ACS Catalysis* **9,** 442-455 (2018).

14. Ren, F.; Zhang, Z.; Liang, Z.; Shen, Y.; Wang, X., et al., Fabrication of cobaltous telluride and carbon composite as a promising carrier for boosting electro oxidation of ethylene glycol on palladium in alkaline medium. *J Colloid Interface Sci* **616,** 316-325 (2022).

15. Si, D.; Xiong, B.; Chen, L.; Shi, J., Highly selective and efficient electrocatalytic synthesis of glycolic acid in coupling with hydrogen evolution. *Chem Catal.* **1,** 941-955 (2021).

16. Li, S.; Lai, J.; Luque, R.; Xu, G., Designed multimetallic Pd nanosponges with enhanced electrocatalytic activity for ethylene glycol and glycerol oxidation. *Energ. Environ. Sci.* **9,** 3097-3102 (2016).

17. Qiao, B.; Yang, T.; Shi, S.; Jia, N.; Chen, Y., et al., Highly Active Hollow RhCu Nanoboxes toward Ethylene Glycol Electrooxidation. *Small* **17,** e2006534 (2021).

18. Marinho, V. L.; Antolini, E.; Giz, M. J.; Camara, G. A.; Pocrifka, L. A., et al., Ethylene glycol oxidation on carbon supported binary PtM (M = Rh, Pd an Ni) electrocatalysts in alkaline media. *J Electroanalytical Chem* **880,** 114859 (2021).

19. Kresse, G.; Furthmüller, J. Efficiency of Ab-Initio Total Energy Calculations for Metals and Semiconductors Using a Plane-Wave Basis Set. *Comput. Mater. Sci*. **6**, 15−50 (1996).

20. Kresse, G.; Furthmüller, J. Efficient Iterative Schemes for Ab Initio Total-Energy Calculations Using a Plane-Wave Basis Set. *Phys. Rev. B*. **54**, 11169−11186 (1996).

21. Perdew, J. P.; Burke, K.; Ernzerhof, M. Generalized Gradient Approximation Made Simple. *Phys. Rev. Lett.* **77**, 3865−3868 (1996).

22. Kresse, G.; Joubert, D. From Ultrasoft Pseudopotentials to the Projector Augmented-Wave Method. *Phys. Rev. B.* **59**, 1758-1775 (1999).

23. Blöchl, P. E. Projector Augmented-Wave Method. *Phys. Rev. B.* **50**, 17953−17979 (1994).

24. Grimme, S.; Antony, J.; Ehrlich, S.; Krieg, H. J. Chem. *Phys*. **132**, 154104 (2010).
